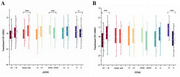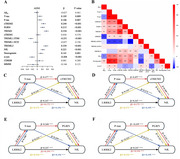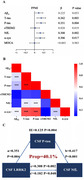# LRRK2 Drives Synaptic Neurodegeneration via Tau Pathology and TREM2‐Dependent Microglial Activation in Alzheimer's Disease

**DOI:** 10.1002/alz70855_101030

**Published:** 2025-12-23

**Authors:** Zehu Sheng, Ming Chen, Jin Zhang, Lan‐Yang Wang, Weihua Yu, Yang Lü

**Affiliations:** ^1^ Department of Geriatrics, The First Affiliated Hospital of Chongqing Medical University, Chongqing, Chongqing, China; ^2^ The First Affiliated Hospital of Chongqing Medical University, Chongqing, Chongqing, China; ^3^ Institutes of Neuroscience, Chongqing Medical University, Chongqing 400016, China, Chongqing, Chongqing, China; ^4^ Department of Psychosomatics and Psychiatry, Zhongda Hospital, School of Medicine, Southeast University, Nanjing, Jiangsu 210096, China, Nanjing, Jiangsu, China; ^5^ Chongqing Medical University, Chongqing, Chongqing, China

## Abstract

**Background:**

Leucine‐rich repeat kinase 2 (LRRK2) is expressed in neurons and glial cells, but the potential role of LRRK2 in the progression of Alzheimer's disease (AD) and its impact on neuroinflammatory pathways remains unclear.

**Method:**

Respectively, 716 and 87 participants from the Alzheimer's Disease Neuroimaging Initiative (ADNI) and Parkinson's Progression Markers Initiative (PPMI) cohorts were included. Participants had cerebrospinal fluid (CSF) LRRK2 or *LRRK2 (rs34637584 and rs76904798)* genotypes, CSF biomarkers for AD core pathology, microglial activation and axonal injury, and underwent cognitive assessments. Statistical models, including one‐way analysis of covariance, multiple linear regression models and Spearman partial correlation analyses, linear mixed‐effects models, mediation analyses, and least squares structural equation modeling were used to explore the role of LRRK2 in the AD pathology, neuroinflammation, and neurodegeneration.

**Result:**

Among participants, the positive tau pathology group had higher CSF LRRK2 levels than the negative group both in ADNI (*p* = 0.017) and PPMI (*p* < 0.001). Moreover, CSF LRRK2 level was significantly correlated with the levels of CSF phosphorylated tau_181_ (*p*‐Tau_181_) (ADNI: β= 0.106, *p* =  0.007; PPMI: β= 0.351, *p* =  0.004), total Tau (t‐Tau) (ADNI: β= 0.105, *p* =  0.009; PPMI: β= 0.362, *p* =  0.003), and NfL (ADNI: β= 0.221, *p* < 0.001; PPMI: β= 0.308, *p* =  0.002). Further, significant indirect effects were found in the TREM2‐dependent mediation pathway in ADNI, CSF LRRK2 → CSF T‐tau or *p*‐Tau → CSF TREM2 → CSF sTREM2→ CSF NfL (IE = 0.013, *p* = 0.036 or 0.032). In PPMI, CSF *p*‐Tau partially mediated the correlation between CSF LRRK2 and CSF NfL (proportion = 40.1%, IE= 0.125, *p* =  0.004).

**Conclusion:**

This study suggests that CSF LRRK2 promotes Tau‐associated synaptic neurodegeneration, and TREM2‐related microglial activation may play an important role in this process.